# Digital Self-Interference Cancellation for Asynchronous In-Band Full-Duplex Underwater Acoustic Communication

**DOI:** 10.3390/s18061700

**Published:** 2018-05-24

**Authors:** Gang Qiao, Shuwei Gan, Songzuo Liu, Lu Ma, Zongxin Sun

**Affiliations:** 1 Acoustic Science and Technology Laboratory, Harbin Engineering University, Harbin 150001,China; qiaogang@hrbeu.edu.cn (G.Q.); ganshuwei@hrbeu.edu.cn (S.G.); malu@hrbeu.edu.cn (L.M.); sunzongxin@hrbeu.edu.cn (Z.S.); 2 Key Laboratory of Marine Information Acquisition and Security (Harbin Engineering University), Ministry of Industry and Information Technology, Harbin 150001, China; 3 College of Underwater Acoustic Engineering, Harbin Engineering University, Harbin 150001, China

**Keywords:** asynchronous in-band full-duplex communication, underwater acoustic communication, nonlinear distortion, sparse constraint

## Abstract

To improve the throughput of underwater acoustic (UWA) networking, the In-band full-duplex (IBFD) communication is one of the most vital pieces of research. The major drawback of IBFD-UWA communication is Self-Interference (SI). This paper presents a digital SI cancellation algorithm for asynchronous IBFD-UWA communication system. We focus on two issues: one is asynchronous communication dissimilar to IBFD radio communication, the other is nonlinear distortion caused by power amplifier (PA). First, we discuss asynchronous IBFD-UWA signal model with the nonlinear distortion of PA. Then, we design a scheme for asynchronous IBFD-UWA communication utilizing the non-overlapping region between SI and intended signal to estimate the nonlinear SI channel. To cancel the nonlinear distortion caused by PA, we propose an Over-Parameterization based Recursive Least Squares (RLS) algorithm (OPRLS) to estimate the nonlinear SI channel. Furthermore, we present the OPRLS with a sparse constraint to estimate the SI channel, which reduces the requirement of the length of the non-overlapping region. Finally, we verify our concept through simulation and the pool experiment. Results demonstrate that the proposed digital SI cancellation scheme can cancel SI efficiently.

## 1. Introduction

The underwater acoustic (UWA) communication and networking are widely used to monitor marine environment and explore ocean resources. However, the throughput of UWA networking is very limited by narrow bandwidth of UWA channel, which is only tens of kilohertz [1,2,3,4,5,6,7]. Thus, research should be done to improve the efficiency and flexibility of the UWA spectrum. In [8], a Full-Duplex (FD) cooperative diversity scheme is proposed, the cooperating nodes receive feedback bites from the destination node about channel state information, which is used to obtain cooperative diversity. The linear detector and interference cancellation detector are also presented. The error rate of the proposed scheme is closed to maximum likelihood detector. In [9], the FD-UWA communication technique with frequency division is used to transmit and receive data simultaneously. However, FD-UWA communication does not increase the spectral efficiency theoretically. Recently, In-band full-duplex (IBFD) radio communication has proven to be feasible and outperform the conventional half-duplex communication in some experiments [10,11]. Thus, it is interesting to investigate the IBFD in the UWA communication system. The major drawback of IBFD communication is the Self-Interference (SI), which is due to simultaneous transmission and reception over the same channel, and the transmitting signal is highly interfered with the receiving signal.

The SI cancellation is generally divided into analog and digital cancellation [12]. The analog cancellation is carried out before Low-Noise Amplifier (LNA) and Analog to Digital Converter (ADC) to avoid the amplitude of receive signal overloading the dynamic of the IBFD communication system. In Ref. [13], the effect of nonlinear distortion occurring in the transmitter power amplifier (PA) and the receiver chain is analyzed, besides the dynamic range requirements of ADC. A measurement-based study of the capabilities and limitations of three key mechanisms for passive analog cancellation: directional isolation, absorptive shielding, and cross polarization is presented in Ref. [14]. In Ref. [15], the analysis of oscillator phase noise effects on the SI cancellation capability of FD direct-conversion radio transceivers is presented, and Closed-form solutions are derived for the power of the residual SI stemming from phase noise in two alternative cases of having either independent oscillators or the same oscillator. The digital cancellation suppresses the residual SI after analog cancellation in the digital field [12,16]. The performance of digital cancellation in the IBFD radio communication system is limited by analog circuit imperfection, such as phase noise of the oscillators, IQ mismatch, the quantization noise of ADC and the PA nonlinearity. Several imperfections in IBFD radio communication have been studied in some literature [13,17,18,19]. In Ref. [17], the phase noise is considered to be a significant limitation of SI cancellation, when independent oscillators are used in the transmission and reception chain. In Ref. [18], the effects of the receiver chain noise figure and the quantization noise are taken into account in SI cancellation. In Ref. [19], the nonlinearities of PA and IQ mismatch are taken into account, and their effects in performance of SI cancellation are also studied. In Ref. [13], the nonlinear distortion is analyzed in detail; analysis results show that, especially with high transmit powers, nonlinear distortion of transmitter PA can significantly contribute to the SI waveform and thus hinder the efficiency of purely linear digital cancellation. Given all of the above, we can draw a conclusion that the main limitations of SI cancellation in the IBFD radio communication system are phase noise, IQ mismatch and nonlinear distortion. However, UWA communication system is different from radio communication system. The carrier frequency of UWA communication is only tens kilohertz, so ADC/Digital to Analog Converter (DAC) can collect/transform the pass band signal directly. The downconversion/upconversion can be completed in the digital domain. It does not need to utilize an oscillator/IQ Mixer to complete upconversion and downconversion, and the carrier signal that is used in upconversion/downconversion is stored beforehand. Therefore, we can assume the carrier signal is perfect and do not study the phase noise and IQ mismatch in this research. We focus on nonlinear distortion and asynchronous problems.

In this paper, we present the system model for asynchronous IBFD-UWA communication, taking into account the effect of the asynchronous communication and nonlinear distortion. The propagation time delay of UWA signal is much longer than the radio signal in air, and this means that SI and the intended signal reach the reception channel at different times; therefore, the IBFD-UWA communication system is asynchronous. For asynchronous IBFD-UWA communication, we propose a novel scheme that utilizes the the non-overlapping region between SI and intended signal to estimate the SI channel. This scheme avoids the effect of the intended signal on SI channel estimation, so this scheme can improve the performance of the SI channel estimation and digital cancellation. The SI signal includes nonlinear distortion, and a nonlinear digital cancellation algorithm needs to be developed. In Ref. [20], the response of nonlinear PA is modeled as a Hammerstein model. In Ref. [21], Ahmed Masmoudi proposed a subspace-based algorithm to jointly estimate the coefficients of both the residual SI and intended channels, and the imperfections of the system including PA nonlinearity are taken into account. However, this algorithm needs more than 70 OFDM symbols to estimate the SI channel. It cannot be used in the UWA system because the UWA channel is quickly time-varying. In Ref. [22], Elsayed Ahmed proposed an iterative technique to jointly estimate the linear SI channel and nonlinearity coefficients. However, this technique needs the training symbol to estimate the linear SI channel before estimating the nonlinear channel. We propose an algorithm based on Over-Parameterization to estimate the nonlinear SI channel. The proposed algorithm translates the nonlinear system to the linear system, and then use the RLS algorithm to estimate the Over-Parameterization parameter. Moreover, we utilize the OPRLS with the sparse constraint to improve the convergence rate of the algorithm and decrease the requirement of the length of the non-overlapping region.

The rest of the paper is organized as follows. In Section 2, we discuss the asynchronous IBFD-UWA signal in detail. In Section 3, we present a novel scheme for asynchronous IBFD-UWA communication and the OPRLS algorithm to cancel the linear SI and nonlinear distortion. Furthermore, we propose the OPRLS with sparse constraint to decrease the requirement of the length of the non-overlapping region. The simulation and experimental results are presented in Section 4. We finally summarize the paper in Section 5.

## 2. System Model

### 2.1. IBFD-UWA Communication System

**Figure 1:**
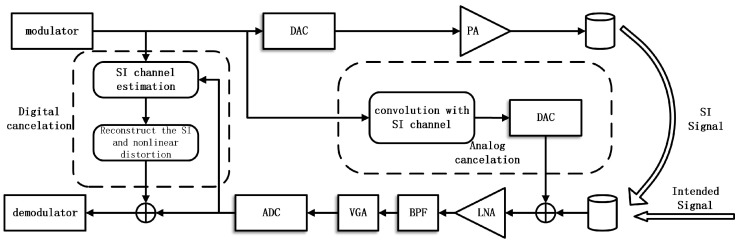
Structure of IBFD-UWA communication system.

The structure of the IBFD-UWA communication system is presented in Figure 1. It can be observed that the model is different from IBFD radio communication [10,12] and UWA half-duplex communication [23]. The architecture that has two transducers is chosen because the transducer is incapable of transmitting and receiving signal simultaneously. Figure 1 shows the two different cancellation stages, and the analog cancellation is performed before the LNA to avoid the overlapping and the digital cancellation after ADC to cancel the residual SI. The Variable-Gain Amplifier (VGA) and Band-Pass Filter (BPF) in Figure 1 is used to amplify and filter the receiving signal. In the analog cancellation stage, we create a signal that is the convolution of the original SI signal with the SI channel in digital domain, and then transform the creating digital signal to analog signal through DAC, and finally subtract the creating analog signal from the receiving signal. The SI channel can be obtained during a short initial half-duplex period. Otherwise, we can observe that we pick the DAC output as the canceler input feeding point, which is different from the analog canceler in Ref. [13]. Therefore, the analog canceler does not cancel the nonlinear distortion caused PA. The reason of using this structure is that, If we choose the PA output, we will need an extra circuit to control the delay and power, and the power consumption of system will increase, the IBFD-UWA communication system, which works for prolonged periods underwater and is powered by battery, is sensitive to power consumption. The digital cancellation is done after the ADC to suppress the residual SI. In this paper, we do not study the analog canceler, and the performance of the analog canceler influences the input intended signal-to-interference power ratio (SIR) of the digital canceler, so we have studied the performance of digital cancellation versus SIR.

### 2.2. The model of Nonlinear Distortion

In this section, we present the model of nonlinear distortion. As Orthogonal Frequency Division Multiplexing (OFDM) is widely applied in the UWA communication system due to its favorable performance [24,25], we choose it as the modulation of IBFD-UWA communication system. The transmitting OFDM signal with N subcarriers in the baseband can be expressed

(1) $$x\left(n\right)=\sum_{k\in S_{A}}s\left[k\right]e^{2j\pi \frac{k}{N}n},$$

where the s[k] is the symbol to be transmitted on the kth subcarrier and $S_{A}$ represents the subcarriers of nonzero symbols. The frequency upshift is done in the digital domain as the center frequency of carrier is only tens kilohertz. We let $f_{c}$ be the center frequency of carrier. The kth subcarrier is located at the frequency

(2) $$f_{k}=f_{c}+\frac{kf_{s}}{N},k=-\frac{K}{2},\cdots ,\frac{K}{2}-1,$$

where the *K* denotes the number of subcarriers, which delivers nonzero symbols. Thus, the passband signal after the DAC can be expressed as

(3) $$\begin{array}{cc} x_{bp}\left(t\right) & =\sum_{k\in S_{A}}s\left[k\right]e^{2j\pi f_{k}t}+\sum_{k\in S_{A}}s^{*}\left[k\right]e^{-2j\pi f_{k}t}\\ & =x\left(t\right)e^{2j\pi f_{c}t}+x^{*}\left(t\right)e^{-2j\pi f_{c}t}, \end{array}$$

where the symbol ${}^{*}$ refers to the conjugate. The PA at the transmitter causes nonlinear distortion. The response of nonlinear PA is modeled with Hammerstein nonlinearity [20]

(4) $$x_{PA}\left(t\right)=\left[\sum_{m=1}^{M}a_{m}x_{bp}^{m}\left(t\right)\right]⨂h_{PA}\left(t\right),$$

where the coefficient $a_{m}$ represents the linear gain and other higher-order nonlinear gain, the $h_{PA}\left(t\right)$ models the memory effect of the PA, and ⨂ denotes the convolution operation. The expansion of $x_{bp}^{m}\left(t\right)$ can be expressed

(5) $$x_{bp}^{m}\left(t\right)=\frac{1}{2^{m}}\sum_{k=0}^{m}\frac{m!}{k!(m-k)!}x^{m-k}{\left[x^{*}\left(t\right)\right]}^{k}e^{2j\pi f_{c}t\left(m-2k\right).}$$

Let the $\mu_{k}=\frac{m!}{k!(m-k)!}x^{m-k}{\left[x^{*}\left(t\right)\right]}^{k}$ , so Equation (5) can be rewritten

(6) $$\left\{\begin{array}{cc} \frac{1}{2^{m}}\sum_{k=0,k\neq (m\pm 1)}^{m}\mu_{k}e^{2j\pi f_{c}t\left(m-2k\right)}+\underset{\mathrm{passband}\;\mathrm{nonlinear}\;\mathrm{distortion}}{\underset{︸}{\mu_{(m-1)/2}e^{2j\pi f_{c}t}+\mu_{(m+1)/2}e^{-2j\pi f_{c}t}}} & \mathrm{if}\;m\;\mathrm{is}\;\mathrm{odd},\\ \frac{1}{2^{m}}\sum_{k=0}^{m}\mu_{k}e^{2j\pi f_{c}t\left(m-2k\right)} & \mathrm{if}\;m\;\mathrm{is}\;\mathrm{even}. \end{array}\right.$$

It can be derived that the transmitting SI signal after PA only contains odd orders of nonlinear distortion in the passband. For a practical communication system, only a limited number of orders contribute to the nonlinear distortion and higher orders can be ignored [17].

### 2.3. The model of Nonlinear Distortion

**Figure 2:**
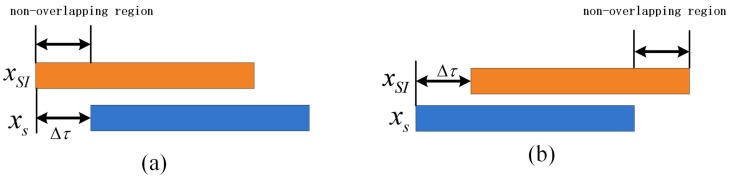
Signal model of asynchronous IBFD-UWA communication system: (**a**) SI is located before intended signal (**b**) SI is located behind intended signal.

The IBFD radio communication system has tacitly assumed that the transmission and reception are synchronized, which means that the SI and intended signal reach the reception chain at the same time. However, in a UWA communication system, the propagation velocity of the acoustic signal in water is about 1500 m/s, and signal propagation delay is even seconds when the distance between different IBFD-UWA communication nodes is thousands of meters. Thus, the SI and intended signal reach the reception chain at the different time, which tells us that the IBFD-UWA communication system is asynchronous. Figure 2 shows the signal model of the asynchronous IBFD-UWA communication system.

Thus, the receiving signal that consists of SI $x_{SI}\left(n\right)$, intended signal $x_{s}\left(n\right)$ and ambient noise $n_{a}\left(n\right)$ after ADC can be expressed

(7) $$\begin{array}{cc} r(n) & =x_{SI}\left(n\right)+x_{s}\left(n-\Delta n_{\tau }\right)+n_{a}\left(n\right)\\ & =x_{PA}\left(n\right)⨂h_{SI\_ch}\left(n\right)+x_{s}\left(n-\Delta n_{\tau }\right)+n_{a}\left(n\right), \end{array}$$

where $h_{SI\_ch}\left(n\right)$ and $\Delta n_{\tau }$ denote the UWA SI channel response and length of non-overlapping region, respectively.

## 3. Digital Cancelation for the IBFD-UWA Communication System

### 3.1. OPRLS for Asynchronous IBFD-UWA Communication System

**Figure 3:**
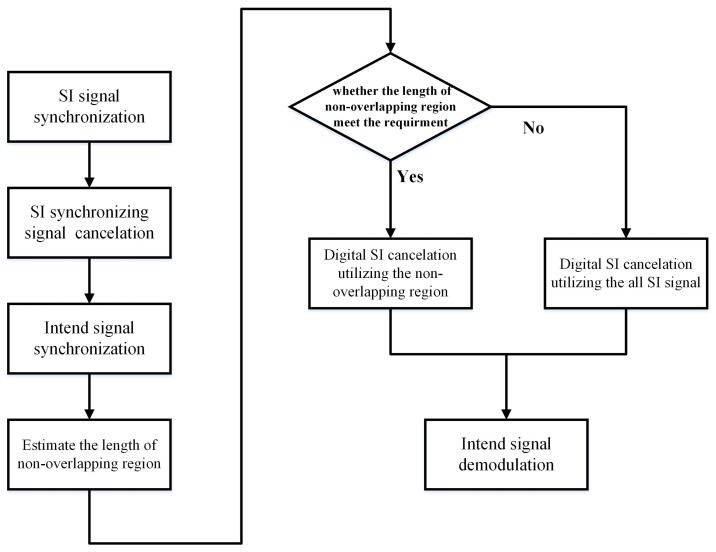
Processing flow chart of digital SI cancellation.

The conventional digital cancellation schemes estimate the SI channel and intended channel at different times, or treat the intended signal as additive noise. For the latter, the intended signal reduces the performance of the digital SI cancellation. For the asynchronous IBFD-UWA communication system, we can utilize the non-overlapping region between SI and the intended signal to estimate the nonlinear SI channel in order to avoid the effect of the intended signal. However, simulation results in Section 4 show that the performance of digital SI cancellation is related to the length of the non-overlapping region; thus, the first step of digital SI cancellation is to estimate the length of the non-overlapping region. In the traditional UWA communication system, the linear frequency modulation (LFM) signal before the data signal is used for synchronization. We do correlation processing to the LFM signal to confirm the position of the LFM and data signal. The phase information that is used in demodulation is obtained from the pilot in the OFDM symbol. In this paper, we adopt different spread spectrum as a synchronizing signal in the various IBFD-UWA communication nodes. The length of the non-overlapping region can be easily estimated through synchronizing SI signal and intended signal. The processing flow chart of digital SI cancellation is shown in Figure 3.

First, we synchronize the SI utilizing the spread spectrum signal, and then cancel the SI synchronizing signal. Furthermore, we synchronize the intended signal using the spread spectrum signal of which spread code is different from SI synchronizing signal. We can estimate the length of non-overlapping region by synchronizing SI and the intended signal. Finally, according to the length of non-overlapping region, we choose to use the non-overlapping region or all SI signals to estimate the nonlinear SI channel.

To estimate the nonlinear SI channel, we present the OPRLS. The non-overlapping region of the receiving signal can be expressed as

(8) $$r\left(n\right)=\left[\sum_{m=1}^{M}a_{m}x_{bp}^{m}\left(n\right)\right]⨂h_{PA}⨂h_{SI\_ch}\left(n\right)+n_{a}\left(n\right).$$

Let $h_{SI}=h_{PA}⨂h_{SI\_ch}$, so Equation (8) can be rewritten as

(9) $$\begin{array}{cc} r(n) & =\left[\sum_{m=1}^{M}a_{m}x_{bp}^{m}\left(n\right)\right]⨂h_{SI}+n_{a}\left(n\right)\\ & =\sum_{l=0}^{L-1}h_{SI}\left(l\right)\sum_{m=1}^{M}a_{m}x_{bp}^{m}\left(n-l\right)+n_{a}\left(n\right), \end{array}$$

where the *L* denotes the length of nonlinear SI channel impulse response and *M* denotes the maximum order of nonlinear distortion. For a more compact representation of (9), we define the set of M×1 vector α and 1×M vector φ(n) as

(10) $$\begin{array}{c} \alpha ={\left[a_{1},a_{2},\cdots ,a_{M}\right]}^{T}\\ \varphi \left(n\right)=[x_{bp}^{1}\left(n\right),x_{bp}^{2}\left(n\right),\cdots ,x_{bp}^{M}\left(n\right)], \end{array}$$

where the symbol ${}^{T}$ denotes transposition. Thus, the receiving signal can be reformulated in vector form from Equations (9) and (10)

(11) $$r\left(n\right)=\sum_{l=0}^{L-1}h_{SI}\left(l\right)\varphi \left(n\right)\alpha +n_{a}\left(n\right).$$

From Equation (11), we need to estimate the L+M coefficients of vector $h_{\mathrm{SI}}$ and α for the Hammerstein model. Based on the over-parameterization principle, we can transform the nonlinear system to a linear system to estimate L×M coefficients. We define the over- parameterization vector c and SI signal vector A(n):

(12) $$\begin{array}{c} c=kron\left(h_{\mathrm{SI}},\alpha \right)={\left[h_{SI}\left(0\right)\alpha ,h_{SI}\left(1\right)\alpha ,\cdots ,h_{SI}\left(L-1\right)\alpha \right]}^{T},\\ A(n)=[\varphi (n),\varphi (n-1),\cdots ,\varphi (n-L)], \end{array}$$

where the kron refers to the Kronecker product between two vectors. Thus, Equation (11) can be rewritten

(13) $$r\left(n\right)=A\left(n\right)c+n_{a}\left(n\right).$$

Assume the length of the non-overlapping region is $\Delta n_{\tau }$. (13) can be reformulated in matrix form as

(14) $$r\left(n\right)=Hc+n_{a},$$

where

(15) $$\begin{array}{c} r={\left[r\left(1\right),r\left(2\right),\cdots ,r\left(\Delta n_{\tau }\right)\right]}^{T},\\ H=[A\left(1\right),A\left(2\right),\cdots ,A\left(\Delta n_{\tau }\right){]}^{T},\\ n_{a}={\left[n_{a}\left(1\right),n_{a}\left(2\right),\cdots ,n_{a}\left(\Delta n_{\tau }\right)\right]}^{T}. \end{array}$$

The least-squares (LS) estimator for the nonlinear channel c can be calculated as

(16) $${\hat{c}}_{LS}={\left(H^{T}H\right)}^{-1}H^{T}r.$$

The Recursive Least Squares (RLS) algorithm can be used to estimate the nonlinear channel instead of LS to avoid evaluating the pseudo-inverse.

### 3.2. OPRLS with Sparse Constraint

**Figure 4:**
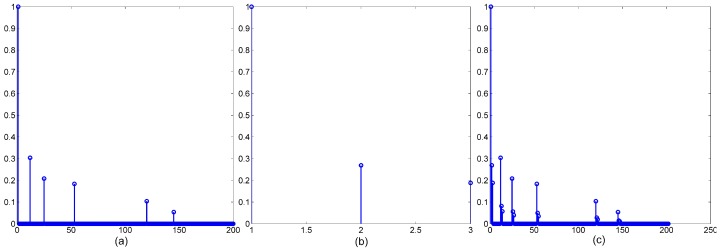
(**a**) UWA channel response $h_{SI\_ch}$; (**b**) PA memory response $h_{PA}$; (**c**) nonlinear channel response $h_{SI}$.

We assume that the length of UWA channel response $h_{SI\_ch}$ is $L_{ch}$ and the length of PA memory response $h_{PA}$ is $L_{PA}$, thus the length of nonlinear channel response $h_{SI}$ is $L_{ch}+L_{PA}-1$. Let $l_{ch}$ and $l_{PA}$ denote the number of nonzero value in $h_{SI\_ch}$ and $h_{PA}$. The total number of nonzero value in $h_{SI}$ is $l_{ch}\times l_{PA}$. The UWA channel is sparse, the $l_{ch}/L_{ch}$ is small and $L_{PA}$ is small too, so the $l_{ch}\times l_{PA}/\left(L_{ch}+L_{PA}\right)$ is extremely small. We can conclude that the nonlinear channel is sparse too. The simulation results in Figure 4 show the UWA channel response, PA memory response and nonlinear channel response.

Since $c=kron(h_{SI},\alpha )$, the c is also sparse. We can utilize the OPRLS with sparse constraint to improve the convergence rate of the algorithm, and decrease the requirement of the length of the non-overlapping region. Firstly, we define the cost function of traditional OPRLS algorithm:

(17) $$\varepsilon \left(n\right)=\frac{1}{2}\sum_{i=1}^{n}\lambda^{n-i}e^{2}\left(i\right),$$

where the λ represents the forgetting factor, and e(i) denotes the error between the expected signal and instantaneous estimate signal

(18) $$e\left(i\right)=r\left(i\right)-A\left(i\right)\hat{c}\left(i\right).$$

Because the nonlinear channel is sparse, we can add the sparse constraint to the cost function of traditional OPRLS algorithm, thus the cost function of OPRLS with sparse constraint can be expressed

(19) $$\eta \left(n\right)=\varepsilon \left(n\right)+\gamma_{s}f\left(\hat{c}\left(n\right)\right),$$

where f(•) denotes the sparse constraint function, and $\gamma_{s}$ represents the regularization parameter. The effect of $\gamma_{s}$ is keeping the balance between the estimate error and sparse constraint in the iteration. We consider the sparse constraint function as $l_{1}$ norm, thus (19) can be rewritten as

(20) $$\eta \left(n\right)=\varepsilon \left(n\right)+\gamma_{s}∥\left(\hat{c}\left(n\right)\right){∥}_{1}.$$

To find the optimal vector $c_{omp}$ , we need to solve $\partial \eta /\partial \hat{c}=0$. Then, we can get

(21) $$\begin{array}{cc} \psi \left(n\right)\hat{c}\left(n\right) & =\Phi \left(n\right)-\gamma_{s}*\left(\partial ∥\hat{c}\left(n\right){∥}_{1}/\partial \hat{c}\right)\\ & =\Phi \left(n\right)-\gamma_{s}*sgn\left(\hat{c}\left(n\right)\right), \end{array}$$

where the ψ(n) represents self-correlation matrix of input signal vector A(n), and the Φ(n) denotes the correlation vector between the r(n) and A(n). The ψ(n), Φ(n) and sgn(∗) can be defined as

(22) $$\begin{array}{cc} \psi (n) & =\sum_{i=1}^{n}\lambda^{n-i}A^{T}\left(i\right)A\left(i\right)=\lambda \psi \left(n-1\right)+A^{T}\left(n\right)A\left(n\right),\\ \Phi (n) & =\sum_{i=1}^{n}\lambda^{n-i}r\left(i\right)A^{T}\left(i\right)=\lambda \Phi \left(n-1\right)+r\left(n\right)A^{T}\left(n\right),\\ sgn(x) & =\left\{\begin{array}{cc} 1, & x>0,\\ 0, & x=0,\\ -1, & x<0. \end{array}\right. \end{array}$$

We define a new vector ρ(n)

(23) $$\rho \left(n\right)=\Phi \left(n\right)-\gamma_{s}sgn\left(\hat{c}\left(n\right)\right).$$

We assume that $sgn\left(\hat{c}\left(n\right)\right)=sgn\left(\hat{c}\left(n-1\right)\right)$, thus (23) can be rewritten as

(24) $$\rho \left(n\right)=\lambda \rho \left(n\right)+r\left(n\right)A\left(n\right)-\gamma_{s}\left(1-\lambda \right)sgn\left(\hat{c}\left(n-1\right)\right).$$

Define the inverse of input signal self-correlation matrix $P\left(n\right)=\psi^{-1}\left(n\right)$. From (22) and the matrix inversion theorem, we can infer the recursion formula of P(n)

(25) $$P\left(n\right)=\lambda^{-1}\left(P\left(n-1\right)-k\left(n\right)A\left(n\right)P\left(n-1\right)\right),$$

where

(26) $$k\left(n\right)=\frac{\left(P(n-1)A(n)\right)}{\lambda +A\left(n\right)P\left(n-1\right)A^{T}\left(n\right)}.$$

Based on (21) and (23), we can get

(27) $$\hat{c}\left(n\right)=P\left(n\right)\rho \left(n\right).$$

We take (24) and (25) to (27), and we can infer the recursion formula of $\hat{c}\left(n\right)$

(28) $$\hat{c}\left(n\right)=\hat{c}\left(n-1\right)+k\left(n\right)e\left(n-1\right)-\gamma_{s}P\left(n\right)sgn\left(\hat{c}\left(n-1\right)\right).$$

The traditional OPRLS recursion formula is different from Equation (28), which summarizes an adaptive sparse constraint.

## 4. Simulation and Experimental Results

**Figure 5:**
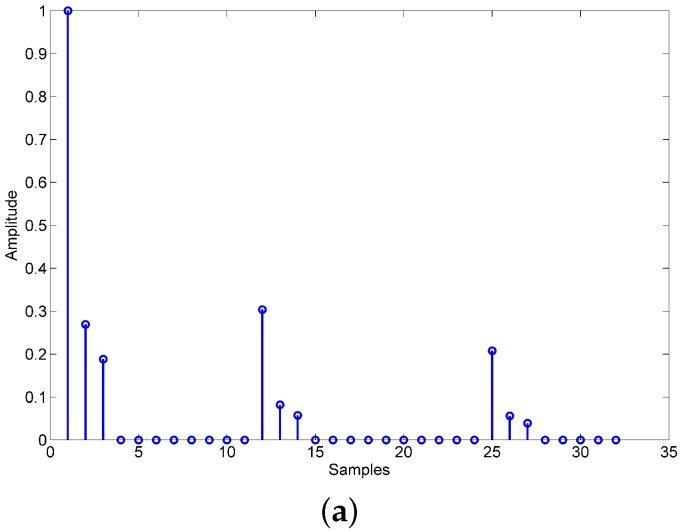

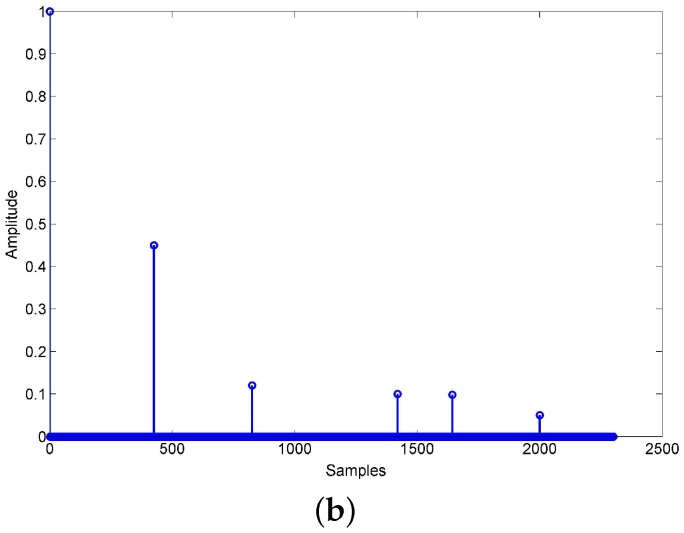
Channel model in simulation (**a**) UWA SI channel; (**b**) intended channel.

In this section, we demonstrate the simulation and experimental results to validate the performance of the proposed algorithm in terms of bite-error-ratio (BER). The nonlinear distortion of PA is realized by the Hammerstein model. The ADC is modeled as a uniform quantization process with the effect of quantization noise. The signal-to-noise ratio (SNR) is the average intended-signal-to-thermal-noise power ratio, and, in simulation, the SNR is 20 dB. In simulation, the UWA SI channel is a measurement model, the Hammerstein parameter of the PA is measured in the laboratory, and the intended channel in the simulation is generated by BELLHOP, which is UWA channel simulation software [16]. The UWA SI channel and intended channel are shown in Figure 5. We utilize an equispaced pilot in the OFDM symbol to estimate the intended channel, the pilot spacing is 3, and the estimation algorithm is LS. Furthermore, the linear interpolation is applied in intended channel estimation. We assume that analog cancellation has been done, and focus on digital cancellation. The parameters describing the utilized OFDM waveform are presented in Table 1.

### 4.1. Simulation Results

**Figure 6:**
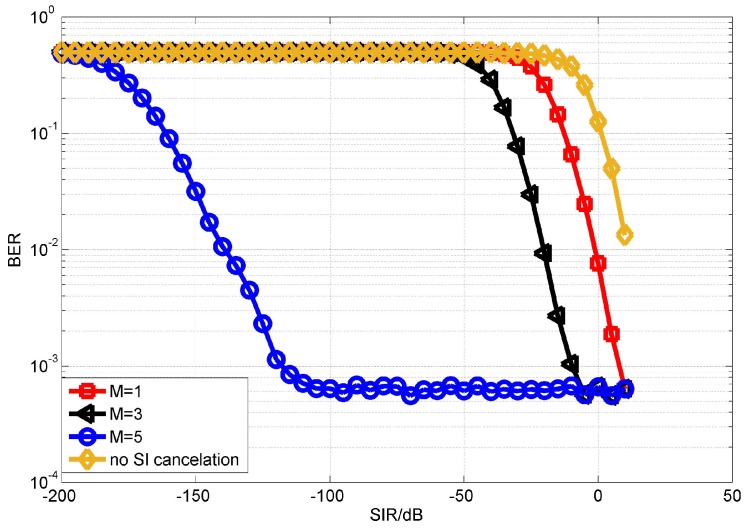
BER performance of OPRLS without quantization noise.

**Figure 7:**
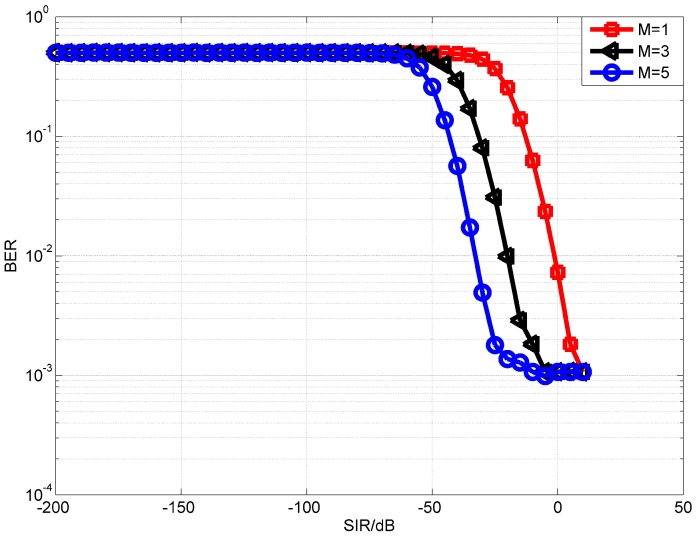
BER performance of OPRLS with quantization noise.

We first evaluate the performance of the proposed OPRLS digital cancellation utilizing all the SI signal. Figure 6 and Figure 7 depict the BER of OPRLS cancellation without and with the quantization noise, respectively. The power of nonlinear distortion caused by PA is 1% of total transmitting signal power. When M = 1, the SI digital cancellation method becomes the same as the traditional linear method. Figure 6 shows that, if any SI cancellation is not applied, the intended signal can not be demodulated correctly when SIR is below 0 dB. The SIR is usually bigger than 0 dB in the practical IBFD-UWA communication system, so we do not show the BER performance of no SI cancellation in the next simulation. The simulation results in Figure 6 and Figure 7 illustrate that the proposed OPRLS algorithm considering nonlinear distortion outperforms the traditional algorithm when SIR is less than −10 dB. Comparing Figure 6 and Figure 7, we can observe that, when SIR is −40 dB to −120 dB, the BER in Figure 7 is much higher than Figure 6. From this phenomenon, we can draw a conclusion that, when SIR is below −40 dB, the main effect of intended demodulation is quantization noise, instead of residual SI. This conclusion is also proved by [13]. In Ref. [13], Korpi et al. show that when transmit power is very large, even perfect linear digital cancellation is not sufficient to maintain the required SINR because quantization noise and nonlinearities become the limiting factors. In this paper, the nonlinearities have been canceled, so the quantization noise is the main factor of getting required SINR.

**Figure 8:**
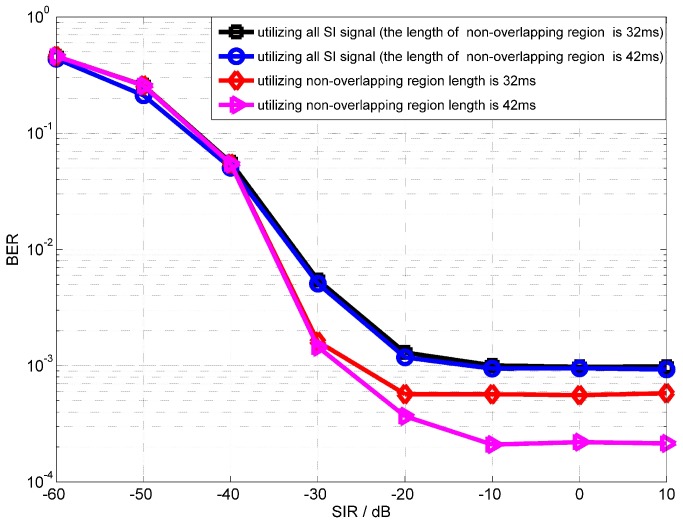
BER performance of OPRLS utilizing non-overlapping region versus SIR.

Figure 8 shows the performance of the proposed scheme that utilizes the non-overlapping region. The length of non-overlapping region in Figure 8 is 32 ms and 42 ms. It is clear that using the non-overlapping region to estimate the SI channel can improve the performance of digital cancellation in an asynchronous IBFD-UWA communication system. Because the proposed scheme avoids the influence of intended signal, by comparing the performance of different lengths of non-overlapping regions, we can draw a conclusion that, for longer non-overlapping regions, a better performance of full-duplex communication can be obtained.

**Figure 9:**
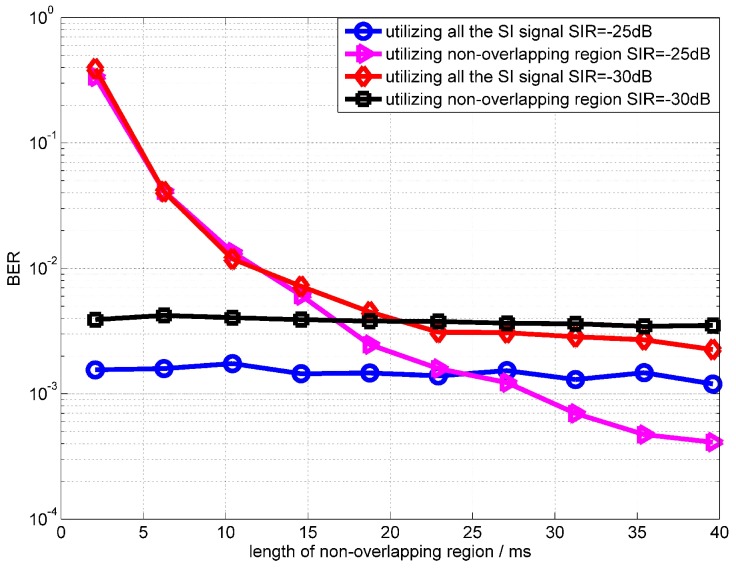
BER performance of OPRLS utilizing the non-overlapping region versus the length of the non-overlapping region.

Figure 9 shows the BER performances at the different lengths of non-overlapping region. The SIR in Figure 9 are −25 dB and −30 dB. From simulation results, it can be found that, when the length of the non-overlapping region is longer than 30 ms, the performance using the non-overlapping region outperforms the algorithm using all SI signals. From Figure 9, we can note that, when SIR is −30 dB, the performance improvement of the proposed scheme is not obvious. Because the power of the intended signal is too low to influence the estimation of the SI channel. For example, the performance of the RLS algorithm is almost the same when SNRs are 30 dB and 50 dB. The simulation results show that the BER performance degrades significantly when the non-overlapping region is less than 10 ms. This number is not related to the signal frequency, and is related to the convergence rate of the OPRLS. Because when the length of the overlapping-region is less than 10 ms, the OPRLS is not convergent enough, the error of SI channel estimation is still large.

**Figure 10:**
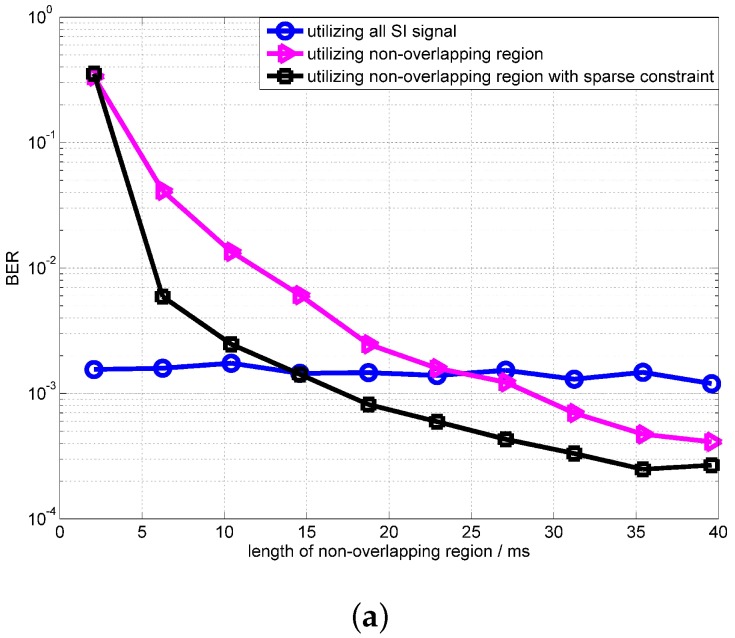

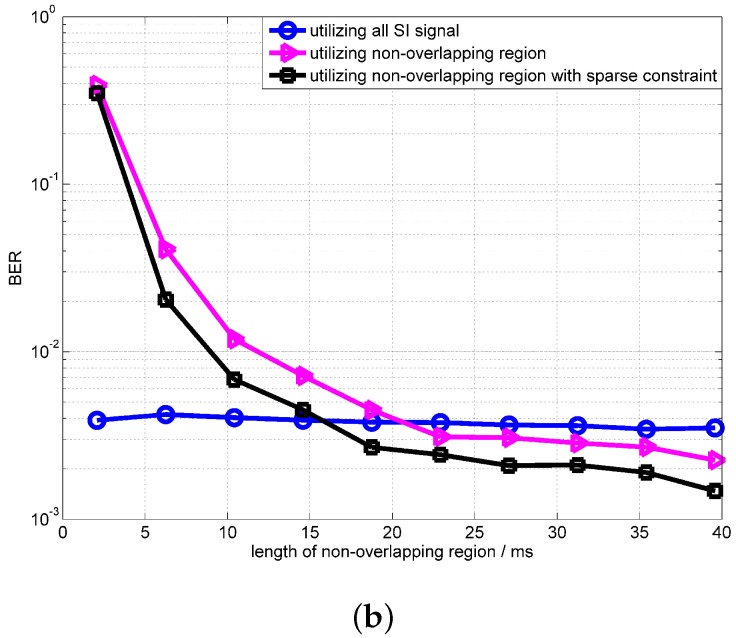
Performance of the OPRLS with sparse constraint (**a**) SIR = −25 dB; (**b**) SIR = −30 dB.

Figure 10 shows the convergence of the OPRLS with sparse constraints. The SIR in Figure 10 is −25 dB and −30 dB. From simulation results, it can be clearly seen that the convergence of the OPRLS with sparse constraints is better than the traditional OPRLS. The BER of the OPRLS with sparse constraints outperforms the algorithm using all SI signals when the length of the non-overlapping region is longer than 20 ms.

**Figure 11:**
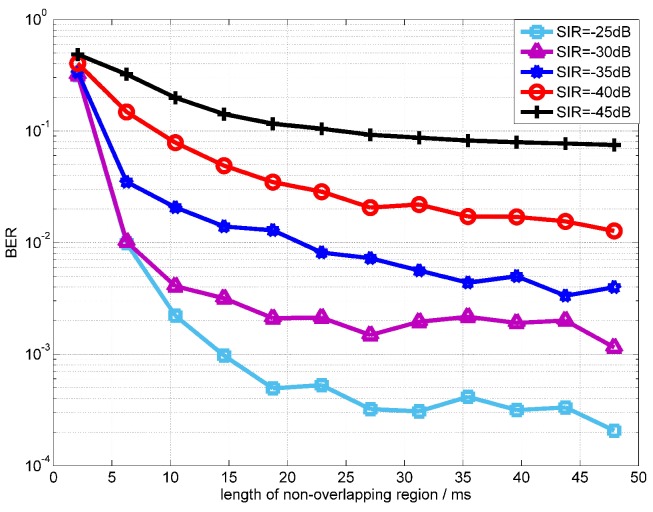
BER convergence performance of the OPRLS with sparse constraints in different SIR.

Figure 11 shows the convergence rate of the OPRLS with sparse constraints in different SIR. The simulation results show that, when the length of non-overlapping is longer than 35 ms, the BER is optimal and the convergence are almost the same in various SIR.

### 4.2. Experimental Results

**Figure 12:**
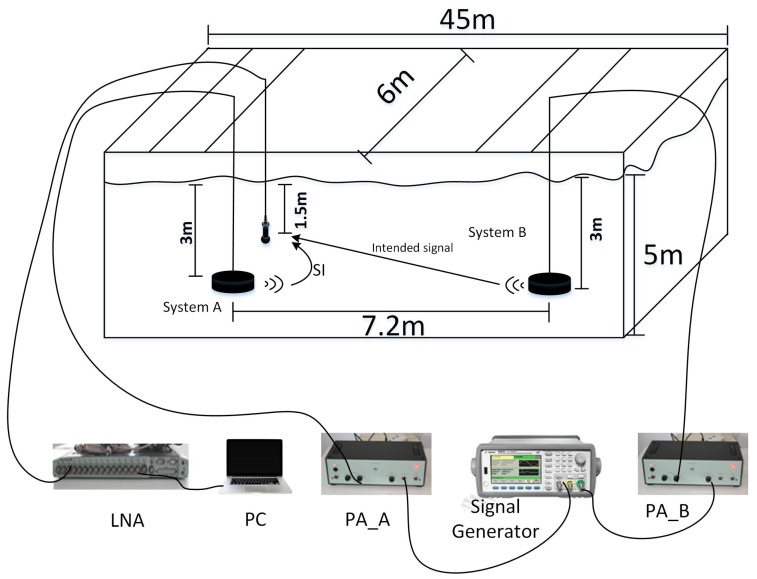
Schematic diagram of a pool experiment.

The experiment was completed in an experimental water pool. Wedge absorbers with a high absorption coefficient are installed in borders and the bottom is filled with sand. The cyclic prefix and cyclic postfix last for 30 ms. We deploy two IBFD-UWA communication nodes A and B with a distance of 7.2 m. The node A has two transducers: one is used to transmit the SI signal and the other is used to receive the signal. For simplification, we only use a single transducer to transmit an intended signal at node B. The depth of the transmitting transducers in nodes A and B are both 3 m, and the depth of the receiving transducer in node A is 1.5 m. We use two-channel function/arbitrary waveform generator 33522A of Agilent to generate SI signal and intended signal, and change the trigger delay of channels to obtain the different lengths of non-overlapping regions. The different SIR is obtained through changing the transmitting power of the SI signal and intended signal. The schematic diagram of the pool experiment is presented in Figure 12. In the experiment, the signal type is the same as the the signal type in the simulation, and nodes A and B transmit different data. The SNR in experiments is 30 dB.

**Figure 13:**
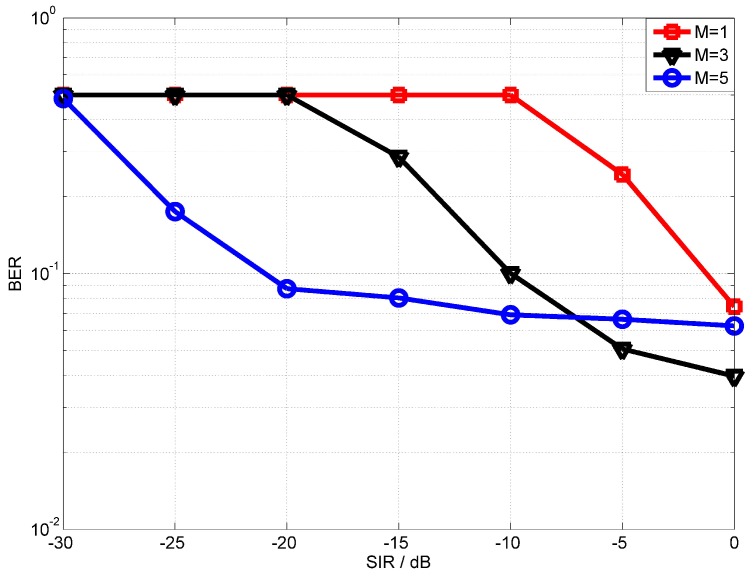
The BER performance of OPRLS.

**Figure 14:**
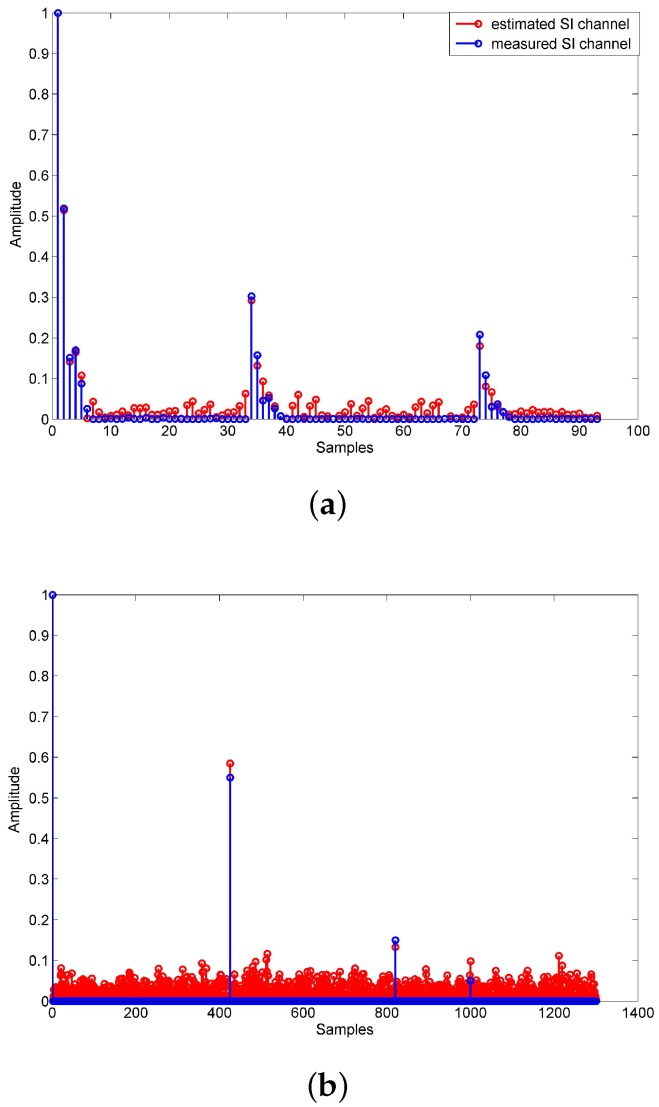
The comparison of measured and estimated SI/intended channel response: (**a**) SI channel; (**b**) intended channel.

Figure 13 shows the performance of the proposed OPRLS digital cancellation using all SI signals. From the experimental results, we can find that the proposed OPRLS algorithm with the nonlinear distortion of PA outperforms the traditional linear digital cancellation. However, when SIR is greater than −10 dB, the BER with M=3 outperforms the M=5. The reason is that, when SIR is higher, the power of higher order nonlinear distortion is weaker than the intended signal, the estimation error of higher order nonlinear parameter increases, and thus the BER degrades. Figure 14 shows the measured and estimated SI/intended channel response when SIR=−15dB. The measured SI/intended channel response is obtained using the training symbol before IBFD communication, so the estimation error is very small.

**Figure 15:**
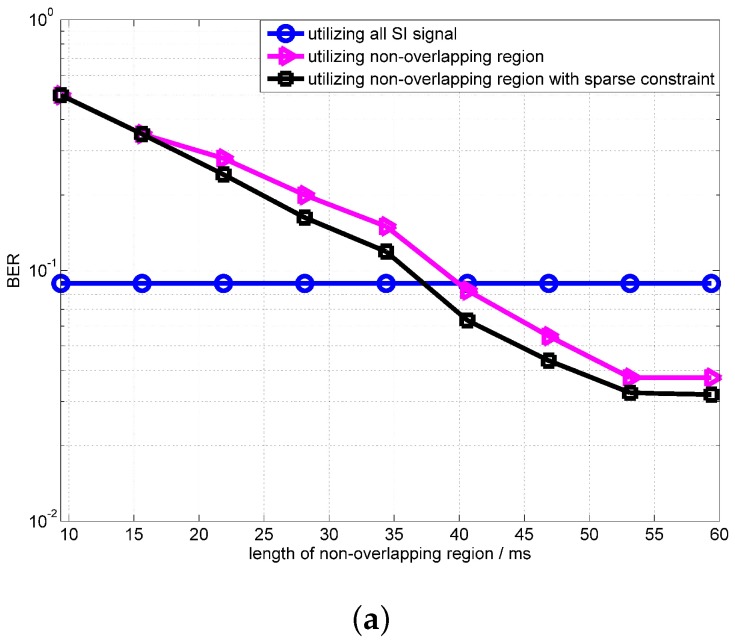

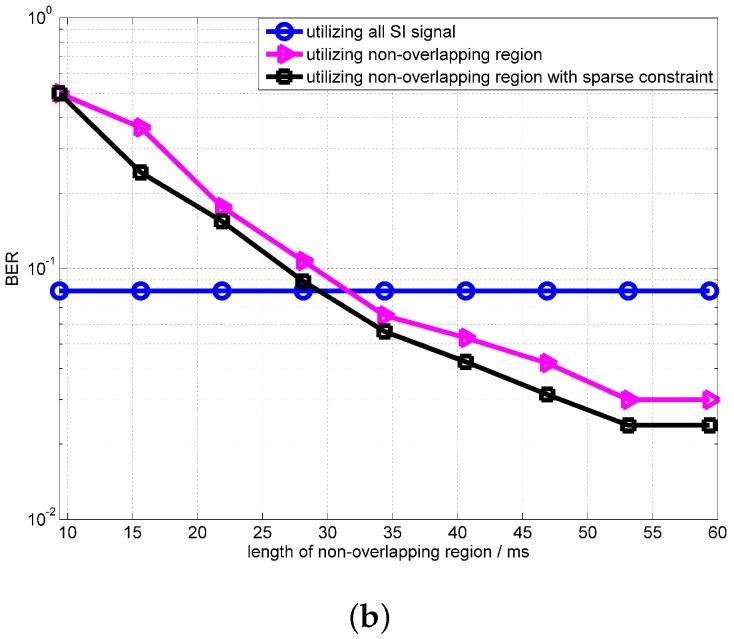
The convergence performance of the OPRLS with sparse constraint (**a**) SIR = −10 dB; (**b**) SIR = −20 dB.

The convergence of the OPRLS with sparse constraints is shown in Figure 15 for various SIR. The experimental results are consistent with the simulation results. The convergence of OPRLS with sparse constraints is faster than traditional OPRLS algorithm without sparse constraints.

## 5. Conclusions

In this work, we firstly propose the signal model of an asynchronous IBFD-UWA communication system. To solve an asynchronous problem in the IBFD-UWA communication system, we present a scheme that utilizes the non-overlapping region to estimate the SI channel. Furthermore, to cancel the nonlinear distortion caused by PA, we put forward the OPRLS with a sparse constraint to estimate the nonlinear channel. The simulation and experimental results show that the BER performance of the proposed scheme outperforms the scheme using all of the SI signal, and the convergence rate of the OPRLS with sparse constraints is faster than the OPRLS. Thus, the OPRLS with sparse constraints decreases the requirement of the length of the non-overlapping region. The research in this paper will be a good candidate for the next-generation communication system, which could improve spectral efficiency and throughput of the underwater network.

## Figures and Tables

**Table 1:** Simulation parameters of OFDM.

Parameter	Value
Constellation	QPSK
Center frequency	12 kHz
Bandwidth	6 kHz
Number of subcarriers	1024
ADC resolution	16 bits
Sample frequency	48 kHz
Pilot spacing	3
